# Parameter estimation for X-ray scattering analysis with Hamiltonian Markov Chain Monte Carlo

**DOI:** 10.1107/S1600577522003034

**Published:** 2022-04-22

**Authors:** Zhang Jiang, Jin Wang, Matthew V. Tirrell, Juan J. de Pablo, Wei Chen

**Affiliations:** a X-ray Science Division, Advanced Photon Source, Argonne National Laboratory, 9700 South Cass Avenue, Lemont, IL 60439, USA; b Materials Science Division, Argonne National Laboratory, Lemont, IL 60439, USA; c Pritzker School of Molecular Engineering, University of Chicago, Chicago, IL 60637, USA

**Keywords:** small-angle X-ray scattering, X-ray reflectivity, Markov chain Monte Carlo

## Abstract

Bayesian-inference-based approaches, in particular the random-walk Markov Chain Monte Carlo (MCMC) method, have received much attention recently for X-ray scattering analysis. Hamiltonian MCMC, a state-of-the-art development in the field of MCMC, has become popular in recent years. It utilizes Hamiltonian dynamics for indirect but much more efficient drawings of the model parameters. We described the principle of the Hamiltonian MCMC for inversion problems in X-ray scattering analysis by estimating high-dimensional models for several motivating scenarios in small-angle X-ray scattering, reflectivity, and X-ray fluorescence holography. Hamiltonian MCMC with appropriate preconditioning can deliver superior performance over the random-walk MCMC, and thus can be used as an efficient tool for the statistical analysis of the parameter distributions, as well as model predictions and confidence analysis.

## Abstract 2

Analyzing X-ray scattering data with the Hamiltonian Markov Chain Monte Carlo method.

## 1. Introduction

Due to the loss of phase information and the impossibility to sample the entire energy/momentum space with sufficient resolutions and absence of errors, many X-ray scattering data analyses are performed through the modeling of relevant processes with physical parameters. The recovery of the model parameters is an inverse problem. Typical optimization procedures for X-ray scattering data analysis attempt to minimize an objective cost function defined for the difference between the experimental data and the model prediction, for example, the sum of squared residuals (SSR) in the least-square regression. Algorithms such as gradient descent, Newton-method, and their variations are often used for parameter optimization in non-linear problems. The parameter uncertainties are often estimated via quadratic approximation, which may not always be reliable in the presence of parameter correlations for high-dimensional problems. Sampling-based Bayesian approaches may tackle these issues, as they offer a statistical insight into the surroundings of the local minima of objective functions, and also facilitate the confidence analysis of parameters and model predictions (Hogg & Foreman-Mackey, 2018 ▸). Taking advantage of recent advancements in statistical machine learning (Bishop, 2011 ▸; Hastie *et al.*, 2016 ▸), Bayesian-inference methods are becoming more popular for analyzing and interpreting X-ray and neutron data. In particular, model parameters can be estimated by the widely applicable Markov Chain Monte Carlo (MCMC) method, which iteratively draws samples with classical random-walk-based Metropolis or Metropolis–Hasting algorithms from the probability distribution of the parameters (Gamerman & Lopes, 2006 ▸). These randomly drawn samples can be used to make statistical inferences, such as uncertainties and confidence levels, about the parameters as well as the variables or functions derived from these parameters for model predictions. While finding the global minimum is not already the primary goal of the MCMC methods, one can eventually reach a solution decently close to the global minimum, if the detailed balance is satisfied and sufficient samples are gathered. Heuristic techniques help during the search for the global minimum or a solution giving physical senses. For example, one may perform a multi-start MCMC with various initial parameters, or narrow the range of initial parameters to be constrained by priors and measurement conditions.

For applications to X-ray and neutron scattering, a few MCMC packages have proved useful. For example, the differential evolution adaptive Metropolis algorithm (ter Braak & Vrugt, 2008 ▸) embedded in *bumps* (Kienzle *et al.*, 2021 ▸) is called by *SASView* (Doucet *et al.*, 2018 ▸) for small-angle scattering analysis, and by *Refl1D* (Kienzle *et al.*, 2011 ▸) for reflectivity analysis. An affine-invariant sampler is another variation of the classical Metropolis–Hasting algorithm based on ensemble sampling (Goodman & Weare, 2010 ▸). It has been implemented in the Python library *emcee* (Foreman-Mackey *et al.*, 2013 ▸, 2019 ▸) called by the *refnx* package for model-based reflectivity analysis of layered films (Nelson & Prescott, 2019 ▸), and in the MATLAB library *gwmcmc* (Grinsted, 2015 ▸) for small-angle neutron scattering analysis of dispersed core-shell nanocrystals (Winslow *et al.*, 2019 ▸). One major challenge of the classical MCMC algorithms is that they are based on random walks and the efficiency of exploring the parameter space may fall off drastically when the dimension of the parameter space increases (Betancourt, 2018 ▸), because the volume of the parameter space becomes more concentrated near the surface region, and thus only a small range of the parameters can be sampled by the classical MCMCs. This is one of the facets of the famous problem known as the curse of dimensionality. The effective sample size (ESS, the number of independent draws) (Vats *et al.*, 2019 ▸) with the classical MCMCs is often small because of the stagnation and high serial correlation (*i.e.* autocorrelation that describes the similarity between random draws as a function of lags; small and rapidly decayed correlations are desired) in the sequence of draws.

The Hamiltonian Markov Chain Monte Carlo (HMCMC) is a recently developed sampling scheme. It attempts to mitigate the inefficient random-walk behaviors in classical MCMCs (Neal, 2011 ▸). Originally introduced as a hybrid Monte Carlo for simulating the lattice field of quantum chromodynamics (Duane *et al.*, 1987 ▸), HMCMC has been greatly advanced and has become one of the most popular MCMC approaches. The principle of HMCMC works in close analogy to the Hamiltonian dynamics. An auxiliary momentum space is introduced and combined with the parameter space to form a state space, and drawing parameters is achieved through the frictionless Hamiltonian dynamics in the state space. The evolution of the dynamics is achieved with numerical integration along the motion trajectory that is fully described by the Hamiltonian equations. It has been well shown that the HMCMC delivers superior performance over the classical MCMC methods for high-dimensional problems because the momentum space facilitates large jumps to distant locations in the parameter space, thus reducing the chance of being trapped in local minima as well as the serial correlation along the draw sequence. Also, the acceptance rate of each jump is relatively high, owing to the energy conservation of the frictionless dynamics. However, this high efficiency of HMCMC is achieved at the cost of computing resources spent on calculating the gradients of the potential energy defined for the parameter space. Fortunately, the gradients can often be calculated either numerically or with automatic differentiation algorithms on modern computers nowadays.^1^ In addition, the accuracy and speed of the numerical integration depend on the magnitude of the gradients, and the discrete evolution step size ε for the integration (equivalent to the discrete-time element d*t* for a time integral). The Hamiltonian dynamics algorithm has been implemented in some packages, for example *pymc* (Salvatier *et al.*, 2016 ▸), *Stan* (Carpenter *et al.*, 2017 ▸), and *LaplacesDemon* (https://web.archive.org/web/20150206004624/http://www.bayesian-inference.com/software). Despite some applications of MCMC to X-ray and neutron data analysis (Sunday *et al.*, 2015 ▸; Fancher *et al.*, 2016 ▸; Metz *et al.*, 2018 ▸; Nelson & Prescott, 2019 ▸; Winslow *et al.*, 2019 ▸), the more efficient Hamiltonian MCMC has not yet been attempted in the field. In the present paper, we will give a quick review of the basic HMCMC to establish notations, followed by the working principles of the HMCMC. We will also describe practical details of preparing the problems required for the HMCMC settings. It will be followed by a few motivating examples in small-angle X-ray scattering (SAXS) and X-ray waveguide fluorescence holography (XWFH) data analysis to demonstrate the performance of the HMCMC.

## 2. Methods

### 2.1. Introduction to Hamiltonian MCMC

Here we will briefly introduce the concept of Hamiltonian Markvo Chain Monte Carlo. Comprehensive reviews can be found elsewhere (Neal, 2011 ▸; Betancourt, 2018 ▸; Fichtner, 2021 ▸). In classical MCMC methods, unknown parameters are randomly drawn from their probability density function 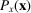 , where 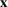 = 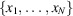 is the set of *N* parameters. In the context of the HMCMC, parameter set 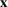 corresponds to the generalized coordinates in classical mechanics, and they will be drawn indirectly through Hamiltonian dynamics. The potential energy of the Hamiltonian system is defined as the negative logarithmic probability density, 

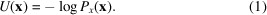

A generalized *N*-dimensional momentum vector 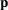 = 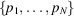 is introduced as an auxiliary variable and the kinetic energy is defined as 

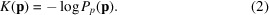

Here 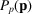 is the probability distribution of the momentum, which often assumes a multivariate normal function, 

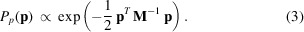

Here, the covariance matrix 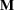 is a symmetric, positive-definite ‘mass matrix’. While an identity matrix 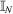 is often assumed, preconditioning with a non-trivial 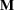 is required for HMCMC to be efficient. With the Hamiltonian expressed as 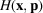 = 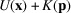 , the joint distribution of the system in the state space 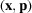 is 

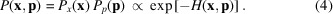

A random draw from this distribution with a classical MCMC is equivalent to the motion of a friction-free system whose dynamics is governed by the Hamiltonian equations 

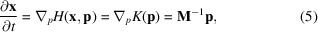




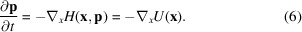

The evolution of the Hamiltonian system is practically achieved via a numerical integration along the trajectory of motion, for which the leapfrog integrator is often applied (Neal, 2011 ▸). Specifically, at coordinate 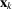 in the parameter space, a state 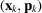 is constructed with a randomly drawn momentum 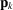 using equation (3). Assigning this state as the initial state 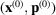 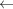 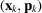 , we can move the system to a new state 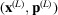 by leapfrogging *L* times with step size ε. At the *l*th step, where 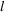 = 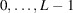 , the integrator is 

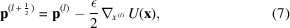




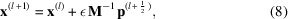




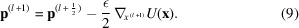

**Figure 1:**
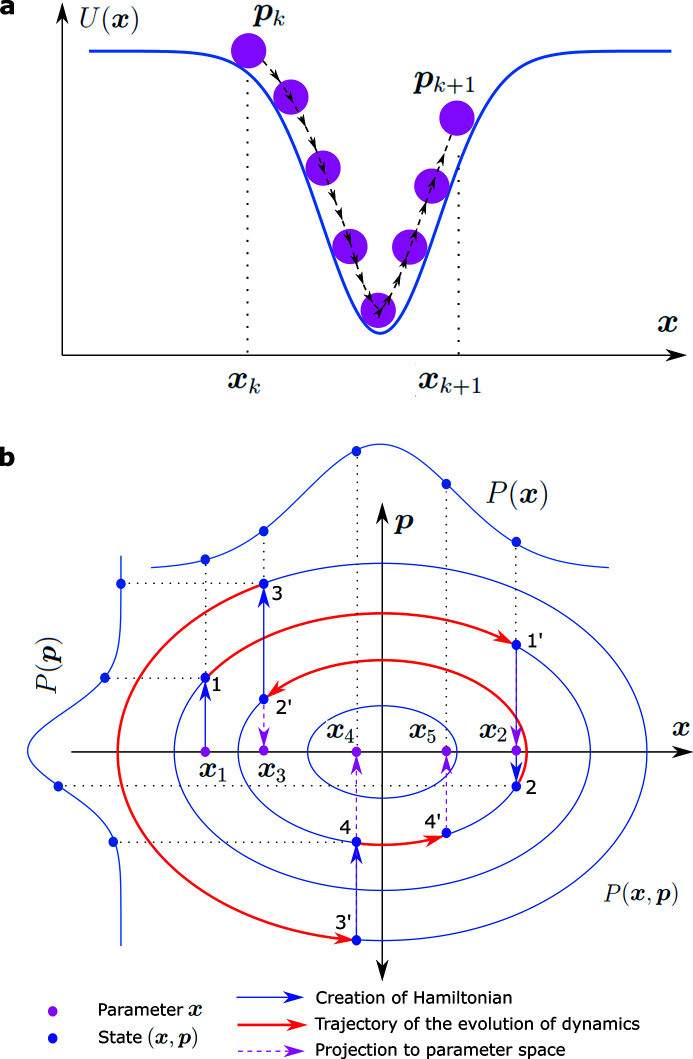
(*a*) Illustration of the evolution of Hamiltonian dynamics to move an object from 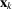 to 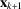 in the parameter space. Flipping the direction of the momentum 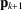 at 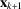 reverses the energy and brings the object back to 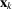 . (*b*) An HMCMC drawing sequence. Contour lines are the joint probability distribution 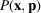 . The topmost and leftmost curves represent the marginal distributions of the parameter and the momentum, respectively. With an initial parameter 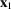 , a random momentum is generated to create a state 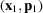 of Hamiltonian 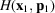 (blue arrow). Parameter 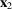 is generated as the result of the evolution from state 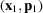 to state 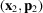 through the trajectory 1 → 1′ (red arrow). This process repeats to generate a sequence of parameters.

After a total integration length Λ = *L*ε, we arrive at a new candidate state 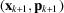 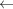 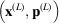 [see Fig. 1 ▸(*a*)]. The property of the friction-free dynamics guarantees the detailed balance required by MCMC because the symmetry of the transition matrix of the Markov chain is naturally ensured by the time-reversibility of the Hamiltonian dynamics, *i.e.* reversing the direction of the momentum vector rewinds the system exactly. The acceptance rate is 100% in theory because of the energy conservation, giving rise to very high efficiency. However, the Hamiltonian is not precisely an invariant, owing to the discretization error of the numerical integration. Therefore, the new state is accepted with a probability 

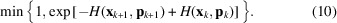

Once state 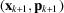 is generated, 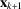 is kept but 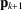 is replaced with another randomly drawn momentum vector to create a new state space with a new Hamiltonian for the next move (Algorithm 1). This iteration continues [Fig. 1 ▸(*b*)] until the Markov chain becomes stationary (a.k.a. burn-in or warm-up), after which a sequence of draws can be collected to estimate parameters and make inferences.

During the evolution, large jumps of 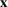 are possibly induced at a high acceptance rate, hence avoiding the inefficient random-walk behavior in the classical MCMCs. With properly selected hyper-parameters 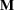 , ε, and Λ, HMCMC may explore a high-dimensional parameter space more efficiently than classical MCMC methods. In addition, HMCMC has a higher tendency to reduce the serial correlation of the sampled sequence (Kass *et al.*, 1998 ▸). While the choice of the hyper-parameters depends on specific inverse problems, the general guidelines have been reviewed elsewhere (Neal, 2011 ▸). 
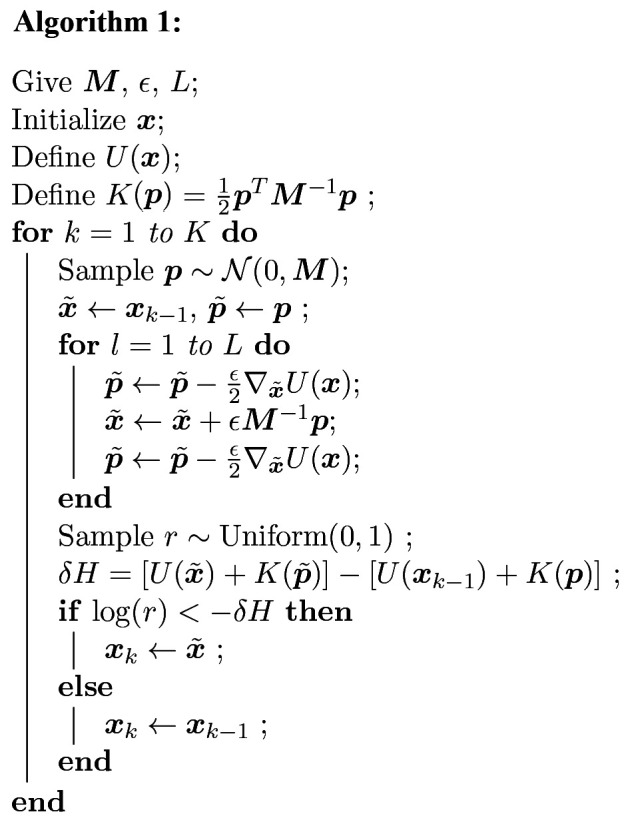


There have also been extensive efforts to optimize the settings of the hyper-parameters in order to further improve the HMCMC’s efficiency. For example, the step size ε can be adaptively tuned on the fly with the dual-averaging algorithm (Nesterov, 2009 ▸; Hoffman & Gelman, 2014 ▸). No-U-Turn Sampler (NUTS) automatically searches for the most efficient integration length Λ to minimize the serial correlation (Hoffman & Gelman, 2014 ▸). Instead of using a fixed step length Λ, NUTS builds a set of candidate states when moving along a path defined by the target distribution and then stops automatically at the location before retracing. The mass matrix also plays an important role in tuning the HMCMC. The sampling of the parameters can be more efficient for linear problems if the inverse of the covariance is used for the mass matrix (Neal, 2011 ▸). In practice, one often collects a number of samples after the sampler reaches stationarity, and then restarts the sampler preconditioned with the inverse of the covariance matrix of samples, as adopted in *pymc* (Salvatier *et al.*, 2016 ▸). This strategy may be challenging in the presence of multi-modals in the target distribution or for high-dimensional models with a band of parameter space that yields acceptable physical interpretations yet impossible for further screening due to experimental condition limitations. An approximated approach is to adopt a form of a scalar multiple of the identity matrix for the mass matrix (Fichtner *et al.*, 2019 ▸; Carpenter *et al.*, 2017 ▸). A recently developed method uses quasi-Newton methods to automatically tune the mass matrix, while keeping the evolution stable with adaptive step sizes (Fichtner *et al.*, 2021 ▸). This method is yet to be explored in the present study. A close inspection of equations (7) and (9) indicates that, if momenta are proposed with 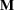 = 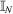 , the dynamics with a single step size ε may be either unstable (too large evolution steps) or too slow (too small evolution steps) in the presence of large variations of the potential gradients 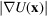 for different parameter dimensions. A practice we used for some examples is to coarsely precondition with a diagonal matrix whose diagonal values are roughly scaled to be proportional to the magnitudes of the gradients computed from samplings obtained from a short warm-up run. This controls the magnitude of the leaps to be incremental and balanced in all dimensions thus increasing the accuracy of dynamics evolution and the speed of convergence. A multi-start strategy with the different initial parameters is also frequently used to avoid falling into local minima and to explore a wider range of the parameter space.

### 2.2. Problem setup for parameter estimation

Parameters are often estimated by minimizing a cost function, which is frequently defined as the SSR between the experimental data and the model prediction (known as the least-square minimization). Maximizing the likelihood of the residuals (known as the maximum likelihood estimation or MLE method), is an alternative method if the residual’s probability distribution is specified. MLE is equivalent to the SSR-minimization if the residual assumes a normal distribution of variance σ^2^, 

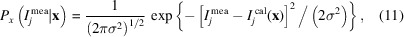

for the *j*th observation data (*j* = 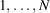 ). Other distributions are applicable, for example Poisson for low-intensity 2D imaging reconstructions (Mario *et al.*, 2018 ▸). Many X-ray scattering data fitting and analysis routines work well by assuming that data points are independently measured and their measurement error distributions are identical. While the error distribution σ_*j*_ can vary point-by-point to reflect the weights of data, observation errors σ are assumed identical here for simplicity and are treated as a separate unknown parameter. We thus can write the total likelihood function as 

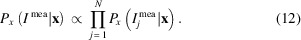

While conventional random-walk MCMC draws samples from this probability distribution, HMCMC does this through the evolution of a Hamiltonian dynamics. With equations (1) and (11), the potential energy becomes 

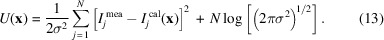

When parameters are bounded or constrained [take 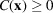 as a general expression], one may transform the parameters to be unbounded or free from constraints in order to help HMCMC search the parameter space. For example, a positive parameter can be reparameterized by a logarithm transformation. Alternatively, one can augment the aforementioned MLE problem by incorporating a prior distribution of the parameters, thus dealing with a maximum a posterior (MAP) problem according to Bayes’ theorem. Thirdly, one may handle the constraints during the sampling such that the conditions are iteratively examined and violating states are simply rejected. However, this approach is inefficient because the decision is made after computing resources have been spent on generating new states that may be immediately discarded. A more efficient way is to check with the constraint during the leapfrogging and reflect the momentum vector without energy loss at the constraint surface 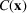 = 0 (Betancourt *et al.*, 2011 ▸).

## 3. Results and discussion

### 3.1. Small-angle X-ray scattering from silica nanoparticles

We first demonstrate the Hamiltonian MCMC on a classical problem: SAXS from dilute spherical silica nanoparticles dispersed in water. The data were taken with 10.9 keV X-rays at Sector 8-ID-I of the Advanced Photon Source (APS), Argonne National Laboratory, USA. The scattering intensity evaluated at a wave vector transfer value *q* is given by 

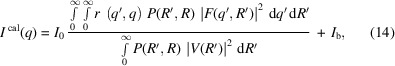

where *I*_0_ is a scaling factor and *I*_b_ is the constant background. *r*(*q*′, *q*), *P*(*R*′, *R*), and *F*(*q*′, *R*′) are, respectively, the resolution function to be convolved (modeled as a normal function centered at each measured *q* with a standard deviation σ_*q*_), nanoparticle size distribution function (modeled as a normal function with mean particle radius *R* and standard deviation σ_*R*_), and the form factor of a spherical particle of radius *R*′, 

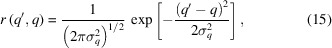




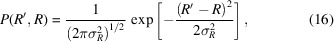




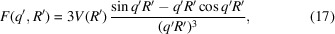

where *V*(*R*) = 4π*R* ^3^/3 is the volume of a particle. The denominator in equation (14) accounts for the scattering volume normalization so that the fraction approaches one as *q* → 0. The parameter space is, therefore, six-dimensional, 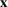 = 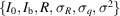 , where σ^2^ is the variance of the residuals of the logarithmic intensity assuming a normal distribution.

**Figure 2:**
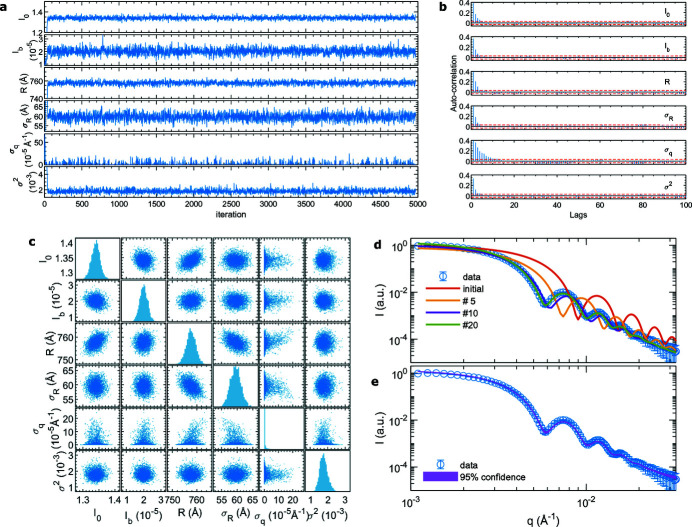
Result of SAXS data analyzed by Hamiltonian MCMC preconditioned with a non-trivial diagonal mass matrix as described in the text. (*a*) Trace of the parameters. The meaning of each parameter is defined in the main text. (*b*) Auto-correlation of the parameters sampled from post-burn-in iterations. The red dashed lines are 95% confidence bounds for the zero auto-correlation hypothesis. (*c*) Probability distributions of the parameters. The off-diagonal panels are joint distributions, and the histograms along the diagonal are marginal distributions. (*d*) Calculated SAXS with parameters from the initial guess, and the 5th, 10th, and 20th HMCMC iterations. (*e*) 95% confidence interval (between the 2.5% and 97.5% percentiles).

Given a well-tuned step size ε and total leapfrog integration length Λ, the speed of convergence is often less of a concern for HMCMC than for random-walked MCMC methods even with far-off initial guesses. Here, the NUTS algorithm (Hoffman & Gelman, 2014 ▸) is used to automatically tune the total leapfrog integration length for the HMCMC. A poor guess, *R* = 500 Å and σ_*R*_ = 20 Å, still leads to a reasonably quick convergence, as seen in Fig. 2 ▸(*a*) where all parameters but the resolution σ_*q*_ become stationary around 50 iterations. To address the concern of being trapped in an undesired local minimum, as a general practice, we carried out multi-start HMCMCs with various initial values and the stationary results were examined in order to filter out nonphysical solutions. In addition, instead of completely random values, the parameters are initialized with sensible values that comply with experiment conditions and prior knowledge about the samples. Fortunately, for examples in the current study, once the model is determined via model assessment and selection (see the next two examples), we did not observe multiple distinct solutions that cannot be ruled out with physical prior knowledge. The speed of convergence is demonstrated in Fig. 2 ▸(*d*), where parameters generated at as early as the 20th iteration can describe the experimental data quite well. We preconditioned the HMCMC for better performance by choosing a kinetic energy such that the mass matrix approximates the inverse covariance of the parameters. A recent advancement worth further exploration has been focused on automatically tuning the mass matrix through quasi-Newton methods (Fichtner *et al.*, 2021 ▸). However, the mass matrix in the present study was approximated from samples of a short run after the warm-up. For further simplicity, we computed the variance of the parameter from a number of pre-run HMCMC iterations and then restarted HMCMC with the mass-matrix set to be the inverse diagonal matrix of the variance. The serial correlation of the sampled parameters quickly decays to beyond the 95% significant level, indicating the efficiency of the HMCMC sampler. The effective sample size determined from the auto-correlation function (ACF) (Neal, 2011 ▸; Fichtner *et al.*, 2019 ▸) is 39% for the mean nanoparticle radius *R*. Joint and marginal distributions of the parameters are shown in Fig. 2 ▸(*c*), and their mean values and standard deviations (not to be confused with the MCMC sampler’s standard errors) are listed in Table 1 ▸. All parameters are over 99% significant, except for the resolution. The exceptionally large *p*-value of σ_*q*_ indicates that the *q*-resolution is perhaps beyond the sensitivity and resolving capability of the data and thus can be removed from the model.

**Figure 3:**
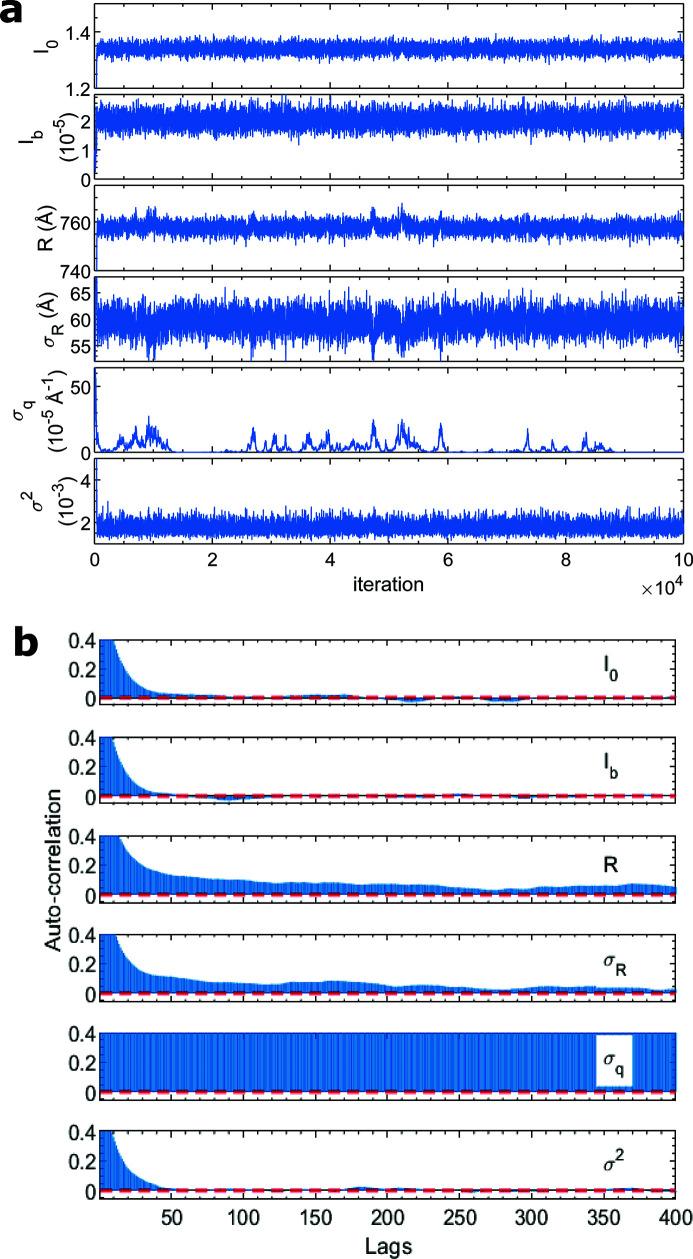
(*a*) Trace of the parameters sampled with random-walk MCMC. (*b*) Auto-correlation of the parameter samplings. The red dashed lines are 95% confidence bounds.

As a comparison, nonlinear least-square fitting was performed. Although it gave a nearly identical mean radius (*R* = 761.3 ± 2.1 Å) and polydispersity (σ_*R*_ = 57.5 ± 1.6 Å), MCMC-based (including HMCMC) methods have a benefit of giving the confidence of the parameters and model predictions [Fig. 2 ▸(*d*)], even in the presence of high correlations among the parameters, as we will see soon in the next example. The conventional random-walk MCMC method was also performed using the multivariate normal distribution for the proposal function with a diagonal covariance matrix determined from the HMCMC result. However, the efficiency of the MCMC is low (Fig. 3 ▸). The ACFs remain high for many lags and the effective sample size is only 2% for *R*, which is undesired for parameter confidence analysis and model predictions. The superior performance of HMCMC may not appear obvious in this toy example when dealing with a small parameter space of only six unknowns. For example, one can run the random-walk MCMC sufficiently long to harvest the same number of absolute effective samples as in the HMCMC. The advantages of HMCMC will manifest in more complex examples of higher dimensional problems as demonstrated below.

### 3.2. Reflectivity from lipid bilayers

**Figure 4:**
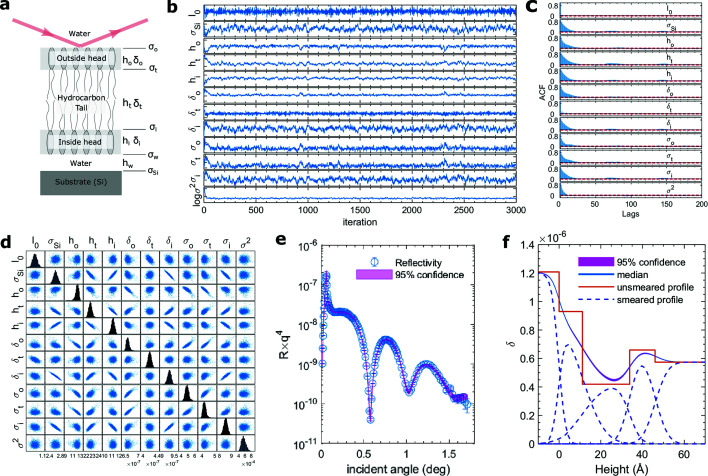
(*a*) Illustration of the reflectivity geometry and the layer parameters in the model. *h*_o,t,i,w_ and δ_o,t,i,w_ are layer thicknesses and dispersions; σ_o,t,i,w,Si_ are interface roughnesses; and *I*_0_ is the incident flux (*i.e.* an intensity scale factor of the reflectivity). (*b*) Traces of parameters from HMCMC. (*c*) ACFs of parameters after 100-iteration burn-in. (*d*) Probability distributions of parameters. The off-diagonal panels are joint distributions, and the histograms along the diagonal are marginal distributions. (*e*) Reflectivity *R* and its 95% confidence from post-burn-in HMCMC samplings. For clarify, the reflectivity is scaled by a factor *q* ^4^, where *q* = 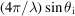 , λ is the X-ray wavelength, and θ_i_ is the incident angle for the reflectivity scan. (*f*) 95% confidence of the dispersion profile. The solid blue line is the median (50%) profile. The red solid line is the step-like profile without roughness smearing. The blue dashed lines are profiles of each layer with smearing.

We now show the application of the HMCMC method to another classical case: X-ray reflectivity from silicon-supported DOPC (1,2-dioleoyl-*sn*-glycero-3-phosphocholine) lipid bilayers in water buffer. The experiment was performed with 20 keV at Sector 1-BM-C at the Advanced Photon Source. The entire bilayer is modeled as consisting of four layers: two hydrophilic head group layers, one hydrophobic hydrocarbon tail layer, and a water cushion layer between the silicon substrate and the the inner side of the head group (Miller *et al.*, 2005 ▸), as shown in Fig. 4 ▸(*a*). In the conventional box model for reflectivity analysis, the roughness is considered as a *q*_*z*_-dependent damping term in a form similar to the Debye–Waller factor (Beckmann & Spizzichino, 1963 ▸; Névot & Croce, 1991 ▸). This method, however, is only valid on well defined layered films where the thicknesses of layers are far larger than the roughnesses of the boundary interfaces. When this *d* ≫ σ criterion is violated, the effective density model is required (Tolan, 1999 ▸; Jiang & Chen, 2017 ▸), where these layer parameters construct a continuous and highly smeared profile in a manner that the component of each layer may penetrate deeply into neighboring layers due to large roughness parameter.

For a quick implementation, a coarse preconditioning is applied with the mass matrix taking a diagonal matrix whose non-zero elements are scaled with the potential gradients. The dual-averaging method was used to optimize the step size ε (Nesterov, 2009 ▸; Hoffman & Gelman, 2014 ▸). With the four-layer model, the thicknesses of the water cushion layer and the inner side head group are found to be *h*_o_ = 3.2 ± 2.1 Å and *h*_i_ = 4.6 ± 2.5 Å, respectively. The two thicknesses are highly uncertain and their correlation coefficient is also high, ρ = −0.987. In addition, the interfacial roughness between the two layers is exceptionally large, σ_w_ = 19.3Å. All this evidence indicates that the water layer and the inner head group should not be treated separately as two distinct layers, and that the experiment data are unable to provide persuasive clues on the existence of the water cushion layer. Instead, with a three-layer model after dropping the water cushion layer, all parameters now become highly significant (Table 2 ▸). The convergence appeared quickly after 100 iterations and the decays of the ACFs of the parameters after the burn-in process display a good independence of the consecutive HMCMC samplings [Figs. 4 ▸(*b*) and 4(*c*)]. The distributions of the samplings also show signs of convergence [Fig. 4 ▸(*d*)]. The 95% confidence level of the constructed density profile (in terms of the dispersion of the index of refraction) is shown in Fig. 4 ▸(*f*) with the corresponding confidence level for the reflectivity in Fig. 4 ▸(*e*). The band of the confidence is narrow, consistent with the narrow distributions of parameters [*e.g.* narrow histograms in Fig. 4 ▸(*d*) and negligible *p*-values in Table 2 ▸ that are numerically calculated from the post-burn-in samplings]. Thus the capability of deriving the correlation between parameters and evaluating confidence of both parameters and models makes this MCMC-based method a powerful tool during model assessment and selection. Finally, as a comparison, we carried out random-walk MCMC with Gaussian proposal functions intialized as the variance of the converged HMCMC result. However, the rejection rate of the MCMC is high, and within a similar computating time we did not obtain a sufficient number of independent samplings to draw conclusions about the models.

### 3.3. X-ray waveguide fluorescence holography for gold-layer depth profiling

**Figure 5:**
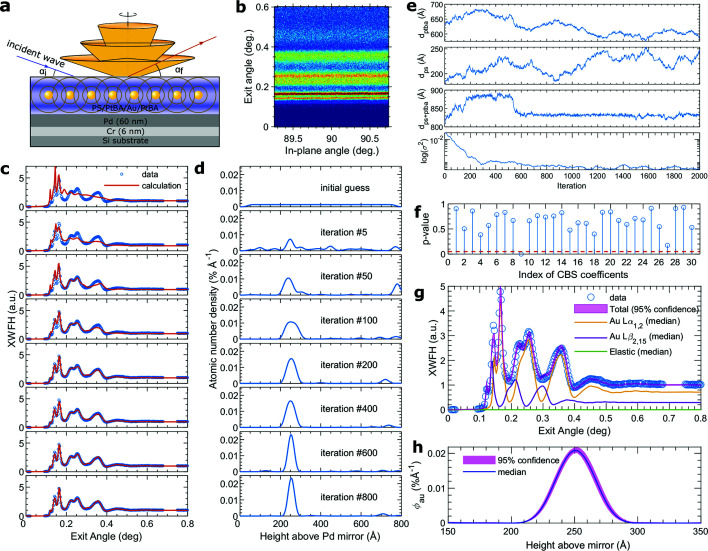
(*a*) Schematics of the thin-film waveguide and principle of the X-ray waveguide fluorescence holography. The meanings of involved parameters to be optimized for are described in the text. (*b*) Fluorescence hologram on an area detector mounted in the plane of the film surface at a right angle with respect to the incident plane. The hologram is integrated in the horizontal plane to obtain the one-dimensional intensity profile as a function of the exit angle α_f_. Panels (*c*) and (*d*) are, respectively, the calculated XWFH and corresponding Au atomic number density profiles at different HMCMC iterations. (*e*) Traces of PtBA layer thickness *d*_ptba_, capping PS layer thickness *d*_ps_, total film thickness *d*_ps+ptba_, and the residual variance. (*f*) *p*-values of the 30 CBS coefficients calculated from the last 1400 iterations. The dashed red line corresponds to a *p*-value of 0.05, *i.e.* a significance level of 95%. (*g*) 95% confidence interval of the total XWFH, and median (50% percentile) contributions from the Au *L*α_1,2_, Au *L*β_2,15_, and elastic scattering background. (*h*) 95% confidence of the Au atomic number density distribution profile. The solid line is the median.

As a general optimization method, HMCMC can also be applied beyond X-ray scattering data analysis. Here we will give a recent example of the application to the depth profiling with the X-ray waveguide fluorescence holography. XWFH is a recently developed high-resolution probe for atom or nanoparticle depth profiling in thin films. While the principle and experimental considerations are described in detail elsewhere (Jiang *et al.*, 2020 ▸), they are briefly illustrated here via an example [Fig. 5 ▸(*a*)]. The goal was to determine the density distribution of a gold nanoparticle monolayer sandwiched between two layers of poly(tert-butyl acrylate) (PtBA) of molecular weight 19.6 kg mol^−1^. This sandwich was capped with a thin layer of polystyrene (PS) to prevent dewetting. The entire film was supported on a silicon substrate pre-coated with a Cr layer followed by a Pd layer. The XWFH experiment was carried out with incident X-rays of 12.1 keV at Sector 7-ID-C of the APS, Argonne National Laboratory. The beam was incident onto the film at a grazing angle of 0.125°, which was between the critical angles of the external reflection for the polymer film and the Pd substrate to enhance the X-ray standing-wave effect as well as to minimize the background scattering from the substrate. At an exciting energy (exchangeable with ‘elastic energy’ below) of 12.1 keV, the gold’s *L*_3_2*p*_3/2_ (11.919 keV) level is excited, giving out two fluorescence groups: *L*α_1,2_ = (9.713, 9.628) keV and *L*β_2,15_ = (11.585, 11.566) keV. These originally isotropic fluorescence emissions are modulated or wave-guided by the thin film, creating two sets of exit-angle-dependent fluorescence cones [termed fluorescence holograms shown in Fig. 5 ▸(*a*)]. The holograms were collected with an area detector mounted in the film surface plane at a right angle (90°) with respect to the X-ray incident plane. Because the fluorescing gold monolayer is isotropic in the plane, *i.e.* no macroscopic in-plane structural modulations, the spatial fluorescence intensity distribution is also isotropic in the surface plane, but it varies as a function of emission/exit angle α_*f*_, thus forming concentric cones. Horizontal lines appear on the area detector as a result of the interception with these cones, as shown in Fig. 5 ▸(*b*). The total measured signals consist of three contributions: two fluorescence holograms corresponding to *L*α_1,2_ and *L*β_2,15_ emissions, and one elastic scattering background. In general, the fluorescence intensity *I*_F_ and the elastic background *I*_E_ are given as functions of the incident and exit angles, α_*i*_ and α_*f*_, such that 

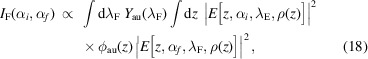




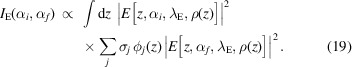

For the fluorescence intensity, λ_F_ and *Y*_au_ are the fluorescence wavelength and atomic yield spectrum of the gold atoms. ϕ_au_(*z*) is the gold atomic number density to be reconstructed, and it is related to the total electron density profile of the film ρ(*z*). The electric field distribution *E* describes the standing wave and it depends on the depth *z*, incident/exit angle, fluorescence/elastic energy, and ρ(*z*). For the elastic scattering background, the contribution arises from the scattering of all atoms in the film and the substrate. The atomic distribution for the *j*th atom type is denoted by ϕ_*j*_(*z*), with its elastic scattering cross-section denoted by σ_*j*_. Because the creation of standing waves here is a result of the dynamical effect in the thin-film waveguide, the reconstruction of the XWFH must employ the dynamical theory to calculate the *E*-field, as is done similarly for the analysis in the grazing-incidence SAXS (Jiang *et al.*, 2011 ▸) and reflectivity (Tolan, 1999 ▸). Also, incident and exit standing waves of different energies must be established to match the two holograms in a self-consistent manner in that they all arise from the same electron density profile ρ(*z*). Therefore, the XWFH with the simultaneous grazing-incident and exit angles imposes very strong constraints on the reconstruction and thus is capable of eliminating the ambiguities often encountered in many other inverse problems.

For generality, the gold atomic number density profile is described with a set of *N*_S_ cubic b-spline (CBS) basis functions, which is often used to construct any arbitrary curve of the second-order derivative continuity (Hastie *et al.*, 2016 ▸), 

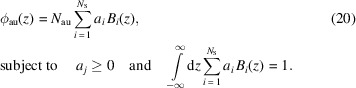

Here, *N*_au_ is the total number of gold atoms and its value corresponds to a nominal layer thickness of pure gold *d*_au_. 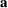 = 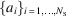 are the CBS coefficients to be reconstructed, and 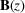 = 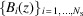 are the CBS basis functions. Model assessment and selection methods help determine the number of splines *N*_S_. In this example, we evaluated models with different *N*_S_ and found that approximately 30 splines (thus 30 unknown spline coefficients) are required to give a sensible result (Jiang *et al.*, 2020 ▸). Assuming no prior knowledge about this profile, we set a constant value for all the initial spline coefficients. A warmup-run indicates that {*I*_0_, *z*_offset_, *d*_ptba_, *d*_ps_, *d*_au_, σ^2^} have higher absolute gradients than other parameters 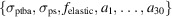 . Here, *I*_0_ is normalization, *d*_ptba_ and *d*_ps_ are the thicknesses of the PtBA and PS layers of corresponding roughnesses σ_ptba_ and σ_ps_, *z*_offset_ is the pixel offset to account for possible mis-calibration of the sample surface plane, *f*_elastic_ is the fractional contribution from the elastic scattering background, and σ^2^ is the residual variance. We thus coarsely preconditioned the HMCMC with diagonal 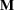 whose nontrivial elements are either 400 or 1. Basic HMCMC is used with fixed evolution step size 0.05 and integration length 0.5 (*i.e.* ten leaps in each iteration).

In spite of no prior knowledge of the gold distribution *(i.e.* flat density), the sign of the convergence of the density profile emerges reasonable fast [Figs. 5 ▸(*c*) and 5(*d*)]. We would like to comment that with our less prudently chosen mass matrix the profile becomes stable at about 600 iterations [shown as the trace of the residual variance in Fig. 5 ▸(*e*)]. One may be able to achieve faster convergence with more careful preconditioning and better parameter initialization. Statistical analysis of 1400 stationary samplings revealed that only one (*a*_9_) CBS coefficient is significantly different from zero at the 95% level [Fig. 5 ▸(*f*) and Table 3 ▸], indicating that the distribution of the gold is well described by a monolayer. Also, we can ignore the contribution of the elastic scattering background because *f*_elastic_ is insignificant. This finding is consistent with two natural experiment configurations of the XWFH technique. First, the detector was mounted at a right angle for minimal elastic scattering. Second, unlike many grazing-exit X-ray fluorescence experiments with a normal incident beam, XWFH was performed with a grazing-incident angle that is below the critical angle of the Pd substrate to reduce the beam penetration into the substrate and thus minimize the background scattering. Therefore, the statistical analysis with HMCMC verified the advantages of the XWFH setup configurations.

In addition, we also noticed that the PtBA/PS interface roughness σ_ptba_ is not significant (*i.e.* large *p*-value), indicating that the PtBA/PS interface is insensible in the data. This is primarily because the scattering contrast between PtBA and PS is negligible. Therefore, the PtBA and PS may be combined as a single layer during the reconstruction, hence reducing the model complexity. This approach is further confirmed by the following two observations: first, *d*_ptba_ and *d*_ps_ are found to be nearly perfectly anti-correlated with a correlation coefficient of −0.992 and their traces move in opposite directions [Fig. 5 ▸(*e*)]; second, the *t*-statistics (Table 3 ▸) reveal that modeling the entire polymer film as a single layer of thickness *d*_ps+ptba_ yields a much higher *t*-stat value (*i.e.* more significant) than dealing with two distinct polymer layers. The trace of the total thickness obviously is much more stable than those of individual layers. Such parameter-correlation analysis and model-complexity assessment represent intrinsic benefits of MCMC methods but they are not readily accessible with direct cost-function minimization or χ^2^ fitting. The confidence intervals for the XWFH and the gold number density profile are very narrow [Figs. 5 ▸(*g*) and 5(*h*)], indicating a high accuracy of the technique and high efficiency of the reconstruction. This performance is due to the intrinsic advantages of the XWFH technique, which combines the profiling information from standing waves of different energies for both the incident and exit angles. For comparison, we also attempted the random-walk MCMC with a multivariate Gaussian proposal function determined from the HMCMC result, but the rejection rate was too high to get it to converge with a reasonable computation time and thus the result is not shown here.

## 4. Summary

HMCMC is still an ongoing research field. There have also been numerous variations and implementations of the HMCMC algorithm. For example, stochastic gradient HMCMC estimates the gradient on randomly selected data subsets from massive data volumes in order to speed up the overall gradient calculation (Chen *et al.*, 2014 ▸). Split HMCMC separates fast and slow movements of the Hamiltonian in the state space of divisible Hamiltonians to lower the overall consumption of computing resources (Shahbaba *et al.*, 2014 ▸). Here, we showed the feasibility of applying Hamiltonian MCMC methods to several classical X-ray scattering cases and demonstrated its performance on parameter estimation, confidence evaluation, and model assessment. While the hyper-parameters for setting up the MCMC and HMCMC are not ideally optimized in the demonstrations, we are still able to observe the advantages of HMCMC in contrast to other conventional methods. High-dimensional model predictions, as well as model building and assessment, may become easier with the Hamiltonian methods even in the presence of parameter correlations. Example data sets and MATLAB (The Mathworks Inc.) scripts for the data analysis are available at the GitHub Repository https://github.com/ennogra/HMCMC or upon request.

## Figures and Tables

**Table 1:** SAXS results with HMCMC

	Mean (s.d.)	*t*-stat	*p*-value
*I*_0_	1.34 (0.01)	97.7	0
*I*_*b*_	2.00 (0.23) × 10^−5^	8.63	0
*R* (Å)	757.7 (2.0)	383.4	0
σ_*R*_ (Å)	59.5 (1.7)	34.6	0
σ_*q*_ (Å^−1^)	1.44 (3.14) × 10^−5^	0.458	0.647
σ^2^	1.78 (0.23) × 10^−3^	7.69	0

**Table 2:** X-ray reflectivity results

	Mean (s.d.)	*t*-stat	*p*-value
*I*_0_	1.06 (0.01)	97.0	0
σ_Si_ (Å)	2.6 (0.1)	33.9	0
*h*_o_ (Å)	12.2 (0.5)	24.8	0
*h*_t_ (Å)	22.7 (0.3)	82.9	0
*h*_i_ (Å)	11.2 (0.2)	51.4	0
δ_o_	6.59 (0.07) × 10^−7^	95.5	0
δ_t_	4.19 (0.05) × 10^−7^	91.6	0
δ_i_	9.30 (0.07) × 10^−7^	133.9	0
σ_o_ (Å)	4.9 (0.3)	17.5	0
σ_t_ (Å)	4.2 (0.1)	31.1	0
σ_i_ (Å)	8.5 (0.2)	54.8	0
σ^2^	5.7 (0.7) × 10^−4^	8.0	0

**Table 3:** XWFH results

	Mean (s.d.)	*t*-stat	*p*-value
*I*_0_	0.122 (0.003)	40.3	0
*z*_offset_ (pixel)	15.71 (0.01)	904	0
σ_air/ps_ (Å)	39.8 (4.6)	8.7	0
*d*_ps_ (Å)	222.2 (15.6)	14.2	0
σ_ps/ptba_ (Å)	25.0 (25.2)	0.99	0.32
*d*_ptba_ (Å)	609.4 (15.8)	38.4	0
*d*_au_ (Å)	7.01 (0.28)	25.4	0
*f*_elastic_	1.46 (6.88) × 10^−9^	0.212	0.83
*a*_9_	9.99 (0.04) × 10^−3^	266	0
σ^2^	0.623 (0.009) × 10^−3^	7.18	0
*d*_ps+ptba_ (Å)	831.5 (3.5)	234	0

## Footnotes

1 More resources can be found at http://www.autodiff.org. The *ADiMAT* (Willkomm *et al.*, 2014 ▸) package was used in this work for gradient computations.
